# Mental health-related stigma discrimination and prejudices among Greek healthcare professionals

**DOI:** 10.3389/fpsyt.2022.1027304

**Published:** 2022-11-30

**Authors:** Georgia-Nektaria Porfyri, Maria Athanasiadou, Vasileios Siokas, Sofia Giannoglou, Sofia Skarpari, Michail Kikis, Artemis Myroforidou, Maria Anoixa, Nikolaos Zerakis, Eleni Bonti, Anastasia Konsta, Ioannis Diakogiannis, Jobst Rudolf, Georgia Deretzi

**Affiliations:** ^1^First Psychiatric Clinic, “Papageorgiou” General Hospital, School of Medicine, Faculty of Health Sciences, Aristotle University of Thessaloniki, Thessaloniki, Greece; ^2^Department of Neurology, University Hospital of Larissa, Faculty of Medicine, School of Health Sciences, University of Thessaly, Larissa, Greece; ^3^Department of Neurology, “Papageorgiou” General Hospital, Thessaloniki, Greece

**Keywords:** stigma, mental health, mental illness, stigma-reduction, healthcare professionals

## Abstract

**Introduction:**

Research shows that mental health-related stigma, stereotypes, and prejudices have a negative impact on the patients themselves as well as on their families and social entourage. Healthcare professionals, whose expertise and professional ethos are historically acknowledged by public opinion, are expected to play a major role in combating discrimination against psychiatric patients. In this study, we aimed to assess the attitudes of Greek healthcare professionals toward mental illness and people suffering from it.

**Materials and methods:**

It is a non-interventional, analytic study, in which 479 health workers from a tertiary hospital in Thessaloniki, Greece, participated. Every single hospital service –except the personnel of the Psychiatric Clinic– was included in our study: from the cleaning service to the administrative staff and the auxiliary staff such as stretcher carriers, food and nutrition services’ staff, and social workers, the nursing staff, and finally the attending physicians, taking into consideration that the psychiatric patient, from the moment he/she enters the hospital, consecutively gets in contact with every work grade of the healthcare establishment. Participants’ attitudes concerning mental illness have been evaluated using the Opinions about Mental Illness Scale (OMI), the Social Distance Scale (SDS), and the Level of Contact Report (LCR-12).

**Results:**

Despite the high level of familiarity [as evaluated with LCR-12; mean score (μ): 8.82 ± 1.73], the employees displayed a rather poor willingness to interact with psychiatric patients (as measured with SDS; μ:11.68 ± 4.28), and endorsed significant prejudice toward individuals with mental disorders (assessed using OMI subscales; Social Discrimination μ: 22.99 ± 12.08, Social Restriction μ: 17.45 ± 9.07, Social Care μ: 21.04 ± 4.12, Social Integration μ: 16.38 ± 4.68, Etiology μ: 9.80 ± 4.95). Age and education stood out as the main determinants of participants’ attitudes, with younger and highly educated participants to have shown a relatively refined profile.

**Conclusion:**

These results are not significantly improved compared to those of previous decades in Greek healthcare professionals and call for critical reflection and targeted stigma-reduction efforts.

## Introduction

The word “stigma” comes from the Greek verb “στ*íζ*ω” *(stizo)* which means “to mark with a scar” (1), and has had, almost timelessly and universally, a negative meaning. In Ancient Greece, slaves were stigmatized, to be distinguished as the lowest in the social hierarchy (2). As Plato quoted in The Laws (page 854d), ‘if anyone is captured performing blasphemy, if he be a slave or a foreigner, let his felony be marked on his visage and his palms.’

According to the American Psychiatric Association’s Dictionary of Psychology, stigma is defined as the dismissive social attitude attached to a feature of an individual that may be considered as a psychiatric, corporal, or communal inadequacy. Stigma involves social disapprobation and can gradually result in unjustified discrimination and rejection (3).

Researchers today categorize mental health-related stigma into three different types: (a) social (public) stigma which involves the negative or discriminatory attitudes that others have about mental illness; (b) self-stigma which refers to the negative attitudes, including internalized shame, that people with mental illness experience about their own condition; and (c) institutional (structural) stigma, which is more systemic and involves policies of government and private organizations that intentionally or unintentionally limit opportunities for the psychiatric patients. Examples of such approaches favoring mental health-related stigma include lower funding for mental illness research as well as poorer access to mental health services compared to other healthcare services (4).

Social stigma that accompanies mental illness has a long-lasting tradition (5) and has been recognized as a serious obstacle to requesting help from mental health professionals (6). Due to mental health stigma, patients face many negative social consequences. Of all groups with chronic conditions or disabilities, they are one of the most unlikely to obtain employment (7), be in a secure and long-lasting relationship (8), have a proper housing (9), and finally experience social integration (10). Furthermore, they often undergo social seclusion, experiencing poor self-confidence and internalized pessimistic thoughts (11).

Social campaigns help to expose these issues as well as to relieve the arising concerns and currently many people identify stigma as a problem (12, 13). Unfortunately, disfavoring opinions still exist, urging mental health patients to refrain from treatment, finally resulting to the worsening of their condition (14, 15).

Public stigma remains a crucial issue also for Greek society. Research examining the existence of mental health-related stigma in the Greek culture showed that Greek citizens carry medium-high level of authoritarian attitudes (the opinion that psychiatric patients are inferior) and a moderate level of social restrictiveness (the opinion that they should be secluded and attentively monitored in the community), despite their high degree of sympathy toward them (16). This finding is in concordance with previous studies, as well (17–19).

Additionally, despite the modern psychiatric reform, stigmatization phenomena are still observed among Greek healthcare providers. A survey conducted in a provincial hospital in Greece revealed that health professionals, although being more confident about the competencies of the psychiatric patients, appear to be biased, confirming that the stigma of mental illness still exists (20). Some previous researchers have reported that younger age, less authoritarian personality characteristics, as well as higher educational and familiarity levels are associated with more positive attitudes toward psychiatric patients among health professionals, while doctors appear to carry fewer stigmatizing notions than other healthcare workers (21) and nurses display contradictory tendencies (22, 23).

Furthermore, in a study conducted in Greek psychiatric rehabilitation centers, health professionals appeared less disposed to adopt a positive attitude toward the treatment of mental illness, to propose amelioration of the offered services’ quality, and to motivate the patients for equal presence and inclusion in the community (24). In a recent study, Greek mental health professionals appeared willing to keep a social distance from people with serious mental disorders, while negative attitudes emerged, including futility of rehabilitation and considering patients as divergent (25). In another study examining Greek mental health professionals’ opinions about psychiatric patients, some stereotypical opinions were documented regarding treatment duration, perceptions of psychiatric patients, and finally probability of recovery (26).

All health professionals are required to treat every patient with the utmost care and understanding, as they are invited to offer their services to individuals who are in a state of vulnerability due to their health condition. Especially regarding persons with mental disorders, health professionals have an additional duty to contribute decisively to the reduction of discrimination, stereotypes, and prejudices against them, both within their professions and society. Firstly, due to the historically significant role that their professions played in the exclusion of these patients from society and secondly because they are perceived by the public as “experts” on these individuals, and their accounts are likely to be believed and respected among members of the general public (27–29). Nevertheless, recent findings support that health –and mental health- professionals should realize and specify their role to a supporting one, by taking a step back and allowing the psychiatric patients to lead this fight, and focus on decisively amplifying these efforts (30). Our study seeks to make an approximate measurement of the presence and degree of stigmatization in the care and the reception of psychiatric patients among the major groups of health professionals in our hospital.

## Materials and methods

### Study design

This is a non-interventional, analytic study, in which 479 employees from “Papageorgiou” General Hospital of Thessaloniki, Greece, participated. “Papageorgiou” General Hospital is a Private Legal Non-profit Entity, established in 1999, in the western part of Thessaloniki, providing preventive care, diagnosis, treatment, and rehabilitation services as well as inpatient and outpatient services. It is fully integrated within the Hellenic National Health System and is on duty according to the on-call schedule of Thessaloniki’s hospitals. The installation of university clinics in 2004 has completed in the best way by the Hospital’s personnel, which has been in constant collaboration with the Aristotle University School of Medicine from that time. According to the hospital’s recorded statistics for 2021, “Papageorgiou” Hospital employs a total of 1.871 individuals; 562 doctors, 924 nurses, and 385 other staff members (31).

After the approval of the study protocol by the Institutional Review Board of “Papageorgiou” General Hospital, the directors of every working unit were informed about and consented to the distribution of the questionnaires to the employees. The personnel of the Hospital’s Psychiatric Clinic (doctors, nurses, psychologists, special educators, speech therapists) were purposely excluded, taking into consideration their specific training, as well as the different level of familiarity with mental disorders, and their exposure to psychiatric patients in more acute and difficult phases. Their exclusion does not assume that they necessarily have improved or worse attitudes, but we considered them as a specific population that would be better examined separately, as a topic of a different study. The purpose of the current research is the evaluation of attitudes toward psychiatric patients from the very first moment they enter the hospital as common citizens, irrespective of whether their altered mental state is known or not (another difference with the psychiatric department, where the presence of mental disorder for the inpatients or outpatients is given or implied).

In every other unit of the health care establishment, printed copies of specific questionnaires (see below for details) were distributed to a random sample of employees, following a short explanation of the study’s goals. The participants provided informed consent and completed the questionnaires anonymously, unattended, with an estimated time of completion of 15–20 min. Subsequently, the questionnaires were collected in the same way that they had been handed out. The participation rate varied in every unit, depending mainly on the number of employees present at the initial briefing. In some departments, there was a minority of health workers that openly ignored or doubted the necessity of the survey.

### Questionnaires/Tools

#### Sociodemographic questionnaire

Participants were asked to provide anonymous demographic information on their age, gender, family status, education, work experience, and profession. Regarding work experience, the following clarification must be made: the field was not limited to the experience gained in the studied hospital, but in general. This implies that some professional categories (such as administrative or auxiliary staff) may have worked in different environments before, probably with a different level of interaction with patients.

#### Opinion about mental illness scale (OMI)

They were also given the Opinions about mental illness scale (OMI) (32). The OMI scale was initially developed by Cohen and Struening in 1959 to evaluate the beliefs of healthcare workers regarding mental disease. The present structure of the OMI scale -which was derived from extensive factor analysis of its initial form of 200 items by more than 8,000 people experienced in mental health- includes 51 declarations displayed via a 6-point Likert-type scale (33). Answers vary from 1 (Fully Agree) to 6 (Fully Disagree). Factor analysis of the 51 items revealed the following five subscales: Factor A: Authoritarianism (A, 11 items): the opinion that people with a mental illness cannot be held accountable for their acts, and that they should be controlled by society, Factor B: Unsophisticated Benevolence (UB, 14 items): an attitude that could be placed between tolerance and pity/compassion; Factor C: Mental Hygiene Ideology (MHI, 9 items): the opinion that mental illness is similar to other illnesses, and that it should receive adequate treatment by specialists; Factor D: Social Restrictiveness (SR, 10 items): the opinion that psychiatric patients should be restricted in some social domains, and Factor E: Interpersonal Etiology (IE, 9 items): the belief that the real cause of a mental illness can be found in problematic interpersonal relations (32, 33).

The Greek OMI version (Supplementary Table 1), standardized for the Greek population by Madianos et al. (17), follows a modified evaluative scheme (Supplementary Table 2), which stresses the following five factors:

• Factor 1: Social Discrimination (SD, 16 items): this factor refers to the distinguishing characteristics of psychiatric patients, who are mainly portrayed as inferior individuals compared to those considered as “normal.” It also includes a latent belief that patients suffering from mental illness need to be treated in an authoritarian way. Example items: “There is something about mental patients that makes it easy to tell them from normal people,” “Psychiatric patients let their emotions rule them while normal individuals think about what to do,” “Although patients discharged from mental hospitals may seem all right, they should not be allowed to marry.”
• Factor 2: Social Restriction (SR, 13 items): It represents the tendency that preventive measures should be taken by the society regarding psychiatric patients. It involves dismissive and compulsive notions about sanctions during or after a psychiatric hospitalization. Example items: “There is little that can be done for patients in a mental hospital except to see that they are comfortable and well fed,” “Anyone who is in a hospital for a mental illness should not be allowed to vote,” “All patients in mental hospitals should be prevented from having children by a painless operation.”
• Factor 3: Social Care (SC, eight items): This factor includes positive opinions regarding the treatment ideology, suggesting amelioration of quality of care and social support. Example items: “Our mental hospitals should be organized in a way to make the patient feel as much as possible as if he is living in his home,” “Psychiatric patients who cannot work because of their mental illness should be given money to live on.”
• Factor 4: Social Integration (SI, eight items): This one depicts the need to encourage equality in social participation and inclusion of psychiatric patients in every aspect of life in the community. Example items: “Many psychiatric patients are capable of skilled work, even if they are somehow mentally disturbed,” “Most people in mental hospitals are not dangerous.”
• Factor 5: Etiology (E, six items): This factor refers to the conceptions about the etiology of mental illness, expressing a tendency to attribute that to the patients’ family. Example items: “If the children of mentally ill parents were raised by normal parents, they would probably not become mentally ill,” “Mental patients come from homes where the parents took little interest in their children.”

For every factor, the final score is derived by summarizing the scores of all the items included and subtracting them from a constant number. Higher scores indicate that the respondent leans more toward the attitude expressed by each factor (23). More specifically, higher scores for factors 1, 2, and 5 indicate more stigmatizing and stereotypical attitudes, whereas higher scores for factors 3 and 4 express more favorable perceptions toward mental illness and patients suffering from it.

The OMI scale has been used globally over decades among health professionals’ categories, as well as in different populations such as undergraduate students, general population, and even psychiatric patients’ relatives (32–35). Additionally, the OMI scale has been widely used in Greece, targeting both the general population (17, 18), and subpopulations such as mental health professionals (25, 26, 36) and students (23, 37–39).

#### Social distance scale (SDS)

Participants were also given the social distance scale (SDS) (40, 41), which consists of seven items (Supplementary Table 3), answered using a 4-point Likert-type scale. Example items: “How willing would you feel to recommend a mentally ill person for a job to someone you know?”, “How willing would you feel to have a mentally ill person take care of your children?” Options for the Greek version that was used vary between 0 (Absolutely Unwilling) and 3 (Absolutely Willing) (42), but the scores were reversed in the statistical analysis, in order to be comparable with the results from the international literature. Total scale scores range between 0 and 21, by summing the individual scores of all responses. This scale measures the social distance the interviewee wants to keep toward a person with a particular condition; in the present study, it measures the distance the hospital staff wants to keep from mental health patients (42, 43), with higher scores representing a greater desire to do so.

#### Level of contact report (LCR-12)

The last questionnaire participants were given was the level of contact report (LCR-12), which is a scale developed by Holmes et al. (43, 44). It is a psychometric self-report test that measures familiarity with mental disorder. LCR-12 consists of 12 phrases/answers (Supplementary Table 4), each one of which corresponds to a specific score (from 1 to 12), depending on the ascending degree of familiarity with mental illness that it represents (45). Example items: “I have never observed a person that I was aware had a mental illness” (rank order score 1), “I have watched a movie or television show in which a character depicted a person with mental illness” (score 3), “I have a mental illness” (score 12). Regarding the completion of the scale, the respondent can choose 1 or more of the 12 statements, in case he/she has experienced them before (23, 44). The final score of each participant is equal to his/her answer with the highest score, that is, of the one representing the highest degree of familiarity (44, 46).

For all questionnaires, the validated Greek version was used (17, 23, 42).

### Statistical analysis

Data were evaluated for deviations from normality by Kolmogorov–Smirnov test. Comparison of means scores at OMI subscales (Social Discrimination, Social Restriction, Social Care, Social Integration, and Etiology), SDS, and LCR between categories in sex (male vs. female), age in years [(a) <30, (b) 31–40, (c) 41–50, (d) 51–60, (e) >60], profession [(f) Physicians, (g) Nurses, (h) Administrative employees – including social workers, (i) Stretcher-carriers, (j) Cleaning services, (k) Food-nutrition services, (l) Other – namely ward assistants, midwives, laboratory assistants, physical therapists, security staff], family status [(m) Married, (n) Divorced, (o) Widower, (p) In relationship, (q) Single], education [(r) Secondary education – SE, (s) Higher-educational institution – HEI, (t) Technological educational institute – TEI, (u) MSc, (v) PhD], years of work experience [(w) 5 years, (x) 5–15, (y) 16–20, (z) 21–26, (@) >26] were performed with parametric tests in case of normal distribution (*t*-test, ANOVA). Otherwise, non-parametric tests were applied (Mann–Whitney *U* test and Kruskal–Wallis test). In case of statistical significance, *post hoc* analyses were carried out, to identify demographic differences between specific groups. The same analysis was performed for items 4, 24, 29, 41, and 51 of the OMI scale. Cronbach’s alpha was also calculated in each subscale of OMI, and SDS and LCR scales to assess the influence of each one on the subscale’s internal consistency. Spearman’s correlation was performed to assess the relationship between subscales of OMI, SDS, and LCR. A significance level of < 0.05 was used for all analyses.

## Results

### Sample characteristics

In total, 479 subjects were recruited and stratified based on gender: 70.6% female, 29.4% male; age: 19.3% < 30 years, 24.3% 31–40 years, 32.5% 41–50 years, 23.4% 51–60 years, 0.6% > 60 years; occupational status: 25.9% physicians, 40.0% nurses, 7.7% administrative employees, and 5.4% stretcher-carriers, 1.0% cleaning services’ staff, 7.9% food and nutrition services’ staff and 11.9% other professions; family status: 56.9% married, 6.4% divorced, 1.0% widowers, 14.6% in relationship, and 21.1% single; education level: 30.2% SE graduate, 22.0% HEI, 26.0% TEI, 17.8% MSc, 4.0% Ph.D. and years of work experience: 27.0% < 5 years, 31.6% 5–15 years, 20.5% 16–20 years, 12.1% 21–26 years, 8.8% > 26 years. Detailed sample characteristics are presented at Supplementary Table 5.

### Cronbach’s alpha

The internal consistency was excellent (>0.7) for the items of Social Discrimination and Restriction OMI’s subscales and SDS, acceptable (0.689) for the items of Etiology, and unsatisfactory for the items of Social Care (0.568), Social Integration (0.564), and LCR (0.594).

### Comparison of OMI subscales

Results are presented in Supplementary Table 6.

#### Social discrimination (SD)

Analysis for mean scores regarding Social Discrimination revealed no statistically significant difference in mean scores for the Social Discrimination based on sex. Statistically significant associations were found for age, profession, family status, education, and years of work experience, with lower (less discriminative) scores for the <30 years, physicians, those in a relationship, those with MSc, and those with less than 5 years of work experience. Nevertheless, all groups demonstrated moderate levels of discriminative attitudes (with their answers varying between “probable agreement” and “probable disagreement” with the given notions), while the aforementioned groups barely stood out in a more positive way.

#### Social restriction (SR)

Analysis for mean scores regarding Social Restriction revealed no statistically significant difference in mean scores for the Social Restriction based on sex, family status, and work experience. Statistically significant associations were found for age, profession, and education, with lower(less restrictive) scores for the 31–40 years, physicians, and those with MSc. However, the majority of all groups (except those > 50 years, auxiliary staff other than administrative personnel, secondary school graduates, widowers, and employees of > 26 years work experience) showed a more distinct disapproval of the restrictive measures considering their mean scores, even though standard deviations remained considerable.

#### Social care (SC)

Analysis for mean scores regarding Social Care revealed no statistically significant difference in mean scores for the Social Care based on sex, and family status. Statistically significant associations were found for age, profession, education, years of work experience with lower (less supporting) scores to be for the <30 years, physicians, those with a Ph.D. and less than 5 years of work experience. This factor is considered to be the one with the most positive impact and greatest accordance among the respondents, as all groups showed distinct positive attitudes toward the need for amelioration of the providence for psychiatric patients (mean scores within the spectrum of “agreement” with the items included), while those with work experience > 26 years crossed the barrier to more definite and positive “waters” (spectrum of “full agreement” with the items), and the stretcher-carriers, other auxiliary staff, those > 51 years old and widowers almost came close, as well. It should be highlighted that in this factor we observe a reversal in the classification of the age-professional-educational and work experience groups, compared to the order shown in the other factors.

#### Social integration (SI)

Analysis for mean scores regarding Social Integration revealed no statistically significant difference in mean scores for the Social Integration based on sex, age, and work experience. Statistically significant associations were found for profession, family status, and education, with higher scores (more positive attitudes toward psychiatric patients) to be found in physicians, widowers, and those with Ph.D. Nonetheless, all groups were reluctant to express a positive opinion (mean scores within the spectrum of “rather agree” with the items included). The only group that managed to score higher (within the spectrum that expresses “agreement”) were those > 60 years old, who represent a mere 0.6% of the sample.

#### Etiology (E)

Analysis for mean scores regarding Etiology revealed no statistically significant difference in mean scores for the Etiology based on sex, family status, and years of work experience. Statistically significant associations were found for age, profession, and education, with lower scores (equivalent to less stereotypical attitudes) for the <30 years, physicians, and those with MSc. The mean scores of all groups revealed the participants’ ambivalence, as they ranged at medium levels (expressing “probable agreement” or “probable disagreement” to the stereotypical notions mentioned).

### Comparison of SDS

Analysis for mean scores regarding SDS revealed no statistically significant difference in mean scores for the SDS based on sex and work experience. Statistically significant associations were found for age, profession, family status, and education, with lower scores—depicting greater willingness to associate with psychiatric patients—for the <30, administrative staff, those in relationship, and those with MSc. The mean scores of all groups were found within the spectrum of “probable unwillingness,” while administrative staff and MSc holders managed to enter into the next—but still unsatisfactory—zone of “probable willingness” to interact with psychiatric patients, with the rest of the groups mentioned above coming quite close. Regarding the individual items, respondents appeared more receptive to having a neighbor with mental illness or introducing a patient to their friends, while they were more negative about having a psychiatric patient as the caretaker or spouse of their children, or as a housemate. Results are presented in Supplementary Tables 3, 6.

### Comparison of LCR

Analysis for mean scores regarding LCR revealed no statistically significant difference in mean scores for the LCR based on sex, age, family status, and work experience. Statistically significant associations were found for profession and education, with higher scores to be for the physicians, and food-nutrition services’ staff, those in relationship, and those with MSc or Ph.D. Respondents appeared more aware and displayed greater sensitivity in this questionnaire, with mean scores of all groups (apart from “other” staff – mean score: 7.70) being over 8.29, where “8” stands for the question “My job involves providing services/treatment for persons with a mental illness,” while the items rated higher than that refer to friends/relatives/family/oneself with mental illness. It is remarkable that 72.7% consider providing services to psychiatric patients as part of their job, 40.1% admitted that a relative of theirs suffers from a mental disorder (item rated as 10), while 5.5% declared that they themselves suffer from a mental illness (highest degree of contact report according to LCR: 12). Results are presented in Supplementary Tables 4, 6. Characteristics and opinions of those who selected the high-scoring items of LCR are reported separately in Supplementary Table 7.

### Spearman correlation

Spearman correlation revealed that Social Discrimination and Social Restriction were positively correlated with SDS and negatively correlated with LCR. That is, being more willing to associate with psychiatric patients, as well as being more familiarized with them leads to less discriminative and restrictive attitudes toward them. Also, being less discriminative and restrictive toward psychiatric patients leads to a greater willingness to interact with them. Etiology was positively correlated with SDS, that is, less stereotyped opinions about the causes of mental illness are associated with greater readiness to interact with them, as well as the reverse. Social Integration was positively correlated with LCR and negatively correlated with SDS, which indicates that greater familiarity with and desire to connect with psychiatric patients is associated with a more supportive ideological stance toward them, regarding their equal social participation, and vice versa. Social Care was negatively correlated with SDS, which means that higher willingness to interact with mental patients corresponds to a higher desire for social support and improved provisions for them, and the opposite conclusion as well. Finally, SDS was negatively correlated with LCR, that is, level of familiarity is directly proportional to willingness to associate with mental patients. Results are presented in Supplementary Table 8.

### Comparison of selected items 4, 24, 29, 41, and 51 of OMI scale

The following questions were selected to be separately described, due to their specific weight in capturing stigmatizing and more problematic attitudes. Results are presented in Supplementary Table 9.

#### Item 4

“*Even if psychiatric patients may seem to be okay, they should not be allowed to get married*”. It is included in the social discrimination factor. Analysis for mean scores regarding Item 4 revealed statistically significant difference in mean scores for the Item 4 based on sex, age, profession, family status, education, and years of work experience, with higher scores (less authoritarian) for males, <30 years, physicians, those in relationship, those that graduated from HEI, and those with less than 5 years of work experience.

#### Item 24

“*It would be foolish for a woman to marry a man who once had a serious mental illness, even if he appeared to be fully mentally restored*”. It belongs to social discrimination items. Analysis for mean scores regarding Item 24 revealed no statistically significant difference in mean scores for Item 24 based on sex. Statistically significant associations were found for age, profession, family status, education, years of work experience with lower (more discriminative) scores for the <30 years, physicians, in relationship, those that graduated from HEI, and with less than 5 years of work experience.

#### Item 29

“*Anyone who is hospitalized in a psychiatric unit should not be allowed to vote*”. It is included among the items of social restriction factor. Analysis for mean scores regarding Item 29 revealed no statistically significant difference in mean scores for Item 29 based on sex, age, family status, and work experience. Statistically significant associations were found for profession and education, with higher scores (expressing less restrictive attitudes) for the administrative employees, and those with MSc.

#### Item 41

“*Most women who have been hospitalized in a psychiatric unit should be trusted to look after children*”. It constitutes one of the social integration items. Analysis for mean scores regarding Item 41 revealed no statistically significant difference in mean scores for Item 41 based on age, family status, and work experience. Statistically significant associations were found for sex, profession, and education with lower scores (more favorable attitudes) for males, administrative employees, and those with PhD.

#### Item 51

“*All patients in psychiatric units should be prevented from having children with sterilization*”. It is indicative of social restriction items. Analysis for mean scores regarding Item 51 revealed statistically significant difference in mean scores for Item 51 based on sex, age, profession, family status, education, and years of work experience, with higher scores (representing less restrictive notions) for males, those 31–40 years, physicians, in relationship, those with MSc, and less than 5 years of work experience.

### Presentation of the OMI items with the extreme mean scores and standard deviations

Mean scores and standard deviations for every item of OMI are presented in Supplementary Table 1. In Tables 1, 2, the items that stood out in the total sample—either by their mean score or their standard deviation—are presented.

**TABLE 1:** Items of maximum and minimum mean scores in OMI analysis.

Items of OMI	Mean score*	Standard deviation
Even though patients in mental hospitals behave in funny ways, it is wrong to laugh about them.	1.32	0.704
Anyone who tries hard to better himself deserves the respect of others.	1.58	0.830
Our mental hospitals should be organized in such a way as to make a patient feel, as much as possible, as if they were living at home.	1.73	0.779

Sometimes mental illness is a punishment for bad deeds.	4.94	1.208
Being hospitalized in a psychiatric clinic is tantamount to failing in real life.	5.10	0.997
The best way to handle patients in mental hospitals is to keep them behind locked doors.	5.33	0.821
		

*Answers rating scale from 1 (Fully Agree) to 6 (Fully Disagree).

**TABLE 2:** Items of maximum and minimum standard deviations in OMI analysis.

**Items of OMI**	Mean score*	**Standard deviation**
Even though patients in mental hospitals behave in funny ways, it is wrong to laugh about them.	1.32	0.704
Our mental hospitals should be organized in such a way as to make a patient feel, as much as possible, as if they were living at home.	1.73	0.779
The best way to handle patients in mental hospitals is to keep them behind locked doors.	5.33	0.821

One of the main causes of mental illness is the lack of moral strength, willpower.	3.35	1.437
If parents loved their children more, there would be less mental illness.	3.61	1.524
Mental illness is an illness like any other.	3.24	1.589
		

As shown above, respondents expressed more absolute opinions in favor of mental health patients, in matters of social care (agreement with the items, expressed by low mean scores) and social restriction (disagreement with the items, expressed by high mean scores), whereas their answers also converged regarding the previous two factors (expressed by low standard deviations).

## Discussion

The aim of the present study was to assess the attitudes of health professionals at “Papageorgiou” General Hospital toward mental illness and people suffering from it. “Papageorgiou” General Hospital is a fully equipped tertiary healthcare facility, located in and providing services to Thessaloniki – the largest city in Northern Greece, and the second largest city in the country. Furthermore, it includes a psychiatric ward, where inpatients come into contact with non-psychiatric health professionals in a variety of ways: e.g., when food is transferred to them, their rooms are cleaned, they are carried to other departments for diagnostic tests or examinations for physical symptoms that may occur, and when they receive useful services from social workers and other administrative staff. As a result, there is a greater level of contact between other healthcare specialties and psychiatric patients, in comparison with other hospitals without a psychiatric department. Therefore, it is of special interest to study the type and extent of the stigmatizing opinions and beliefs held by healthcare professionals that work in the other departments of “Papageorgiou” General Hospital toward people suffering from mental disorders.

Greek studies from the last decades indicate that, after the drastic changes and modernization of mental health services since the 1980s (47, 48), there has been distinct progress in the attitudes of the general population and healthcare professionals toward mental illness. However, it seems that this progress has continued to move along at the very slow pace of the still “under-construction” mental health reform that remains incomplete (18, 48, 49). This discrepancy with the quick development in many other areas of modern society and human needs poses a great challenge to the Greek society.

Overall, our study describes a certain degree of positive attitudes toward psychiatric patients among healthcare workers at our institution. However, it also documents that a higher grade of familiarity and interaction with people suffering from mental illness (as indicated by the high LCR scores in our study) does not guarantee by itself the development of adequate favorable opinions toward those patients.

In the following section, we describe the specific features of stigmatization carried out by each group of healthcare professionals and identify areas for possible intervention. Regarding the different groups of healthcare professionals, we observe that:

• Even though there was a difference between the size of the female and male samples, sex did not affect any of the examined factors.
• Family status did not result in remarkable statistical differences, except for the Social Care factor, Social Distance willingness, and some aspects of Social Integration.
• Years of work experience resulted in statistical differences only with regard to Social Discrimination, while the positive attitudes of those working for more than 26 years regarding Social Care stood out among all groups.
• Younger participants (<40 years old) showed a more favorable attitude to mental illness in the fields of Social Distance, Social Restriction, Etiology, and willingness to interact with patients. No statistical differences based on age were found regarding Social Integration, while Social Care was the only sector where people older than 40 years appeared more supportive.
• Education played a consistent role in all examined factors except for Social Care (alongside “age”). More specifically, as higher educational level increases, attitudes toward mental illness become more and more favorable. We should mention the exception of Ph.D. holders, who do not follow precisely the previous rule, probably given that they were a small sample.
• With respect to profession, physicians and administrative staff presented more deficits regarding Social Care. On the other hand, the staff of the auxiliary services showed greater sensitivity in terms of Social Care, with significant deficits in other fields. It is notable that nurses—that is, the largest group—did not stand out in any of the examined factors and were always bridging the—rather small—gap between the other groups.

Summarizing our results, we can conclude that a high level of contact with patients suffering from mental illness is not necessarily associated with the sufficient willingness to interact with those patients, nor does it reduce decisively the existing prejudices. This finding is consistent with other studies indicating that contact with people suffering from mental disorders could be of help under specific conditions, such as interacting with individuals who are not in the acute phase, in a frame that endorses companionship (between peers considered as equal) and includes common goals and joint efforts (50). In a healthcare environment, it is of critical importance to be able to observe the long-term effect of treatment on psychiatric patients, instead of interacting with those not receiving appropriate mental support, remaining unwell, and spreading a sense of hopelessness (51).

Overall, most of our respondents showed a rather paternalistic, but sympathetic view of psychiatric patients, which reflects a certain degree of favorable attitudes toward them. This finding is supported by other studies, which have shown that when it comes to society’s attitudes toward mental health patients, charitable views tend to prevail and the responsibility to provide the best possible care is recognized by a large majority (52, 53).

Taken together, our results show that physicians and administrative staff—despite the fact that the latter may have possible previous work experience in different sectors and the former might be able to claim to work more often and more closely with patients—did not differ significantly with regard to the majority of the examined factors. The same holds true for the auxiliary staff (stretcher–carriers, food services, and cleaning services staff). We cautiously conclude that age and educational level are the main determinants affecting health professionals’ attitudes toward mental illness. This finding is consistent with other studies as well (17–19, 23). Moreover, it is promising for the new generations and it also opens a perspective for intensive educational efforts aiming at the amelioration of health workers’ attitude toward mental illness.

Of course, our study has certain limitations:

– We report results from a single hospital with specific features, as mentioned above. Nevertheless, we claim that it constitutes a random sample of the Greek National Health System. The hospital staff is recruited with the same criteria that are used for the whole public sector. Furthermore, there are no reports from the literature that state contrary findings.
– Some caution is also warranted, as different healthcare professions were not equally distributed in the sample population. Age distribution among these groups was not equal, as auxiliary staff (food services’ staff, stretcher–carriers, and cleaning services) consists mainly of employees older than 40 years old, with a lower education level. Finally, subjects aged over 60 years old, Ph.D. and widowers constitute small groups, and their results should be assessed cautiously.
– Cronbach’s alpha for the items of Social Care, Social Integration, and Level of Contact Report was unsatisfactory.
– The sample includes employees that might have previously worked in different sectors, such as the administrative and auxiliary staff. However, their potential difference in previous working experience was not captured by the questionnaires, and this could have been a factor leading to a differentiation in attitudes toward psychiatric patients, in comparison with those with experience solely in the health sector.
– The exact response rate was not possible to be determined, due to the way the questionnaires were distributed and recollected.
– Differing from other studies, the staff of the psychiatric clinic was excluded (22, 23).
– The research took place during the COVID-19 pandemic. Thus, we were unable to assess whether personal health concerns, anxiety, and professional fatigue influenced the employees’ responses.

### Possible implications of our study

The results of our study are of clinical, educational, and research interest.

Despite an impressive amount of positive attitudes toward patients with mental illness, our study detects a significant degree of stigmatization among healthcare workers of all professions, although with differences between the distinct subgroups. In clinical practice, the presence of prejudiced notions could negatively affect the way that people suffering from mental illness are treated during their hospitalization. They might become objects of underestimation, leading to underdiagnoses and undertreatment (4, 54–56), with clear and present danger to the patients’ health (57–59). Open stigmatization could discourage psychiatric patients from seeking medical assistance (6, 60), often perceived as weakness or failure (51).

Educational efforts and interventions should aim at increasing the level of empathy, fighting ignorance and its consequent misconceptions, as well as at reducing the accompanying fear of healthcare professionals. As reported in previous studies, appropriate educational programs could lead to the amelioration of perceptions of mental illness and of patients suffering from it (15, 61–64). These procedures should start from the mandatory (basic) educational system but should be continued into the higher stages of public education, with special respect to the institutional education of health professionals. Seminars of lifelong learning and campaigns could complete this educative triangle. In order for these changes to take place, universities, hospitals, and public health institutions are requested to press for adequate political action (65). Nevertheless, it should be highlighted that modern literature rejects a sterile educational approach that could bring about some undesirable negative results (e.g., education focusing on the biogenesis of mental illness, despite taking away the causative “blame” from individuals, has been associated with increased hopelessness for patients’ course and amplification of stigmatizing beliefs) (30, 50, 66). What is proposed is the combination (67) of theoretical educative procedures and contact with people with lived experience of mental illness [either face to face or via technological means (68)], that is, people who can narrate their story of success (30, 66), inspire and directly combat stigma. Among these individuals, health professionals suffering from mental illness are nowadays called upon to play a major role in anti-stigma efforts (30, 66). Our results offer the additional possibility of detecting specific features of stigmatization among the subgroups and give focus to targeted interventions based on them.

Regarding future research, it could be very interesting if staff’s attitudes were reevaluated after the implementation of an educative program, in order to detect any differentiations and assess the followed procedure. Moreover, the undergraduate students’ attitudes could be measured and compared to those of the respective working groups, in order to examine whether younger generations are more romantic and show greater understanding, or whether they are indifferent and ignorant (69, 70). Furthermore, possible differences due to the age gap raise the question of whether daily clinical reality impacts negatively the personnel, or instead leads to an increased level of empathy. Also, it is of interest whether the changes in attitudes are due to greater exposure to mental illness or to professional burnout (51). In addition, the attitudes of the employees working in the primary and secondary health care sector (where there is greater familiarity with the patients) could be assessed and compared with our results. The authors are currently running a study on stigmatizing beliefs in medical students and primary healthcare practitioners.

Taking into consideration that the level of a civilization is indicated by the attitude of society toward its most vulnerable and least favorable members, people suffering from mental disorders should hold a special place in society’s heart and social policy. In a country that has undergone rapid changes in the past decades, stigmatization phenomena stemming from lack of awareness and education should be fought. Even though the presented numbers demonstrate a level of amelioration in terms of discrimination, restriction, and etiology, compared to those of previous decades (17–19, 21–23), these changes are insufficient. Numbers call for action, human lives call for understanding, and societies and especially health professionals should remain alert. The Greek health and mental health system’s reform should concentrate not only on the improvement of materials, techniques, and infrastructures, but equally aim at the improvement and refinement of ways and attitudes when providing services to each patient. Even though asylums like Leros are now a thing of the past (47, 71), many actions are still required (48, 72) to replace the flawed present depicted in our study with a future characterized by a decisively positive attitude toward mental illness and the people suffering from it.

## Data availability statement

The original contributions presented in this study are included in the article/Supplementary material, further inquiries can be directed to the corresponding authors.

## Ethics statement

This study was conducted according to the guidelines of the Declaration of Helsinki and approved by the Institutional Review Board of the Neurology Clinic, “Papageorgiou” General Hospital of Thessaloniki, Greece (protocol code: 120/date of approval: 19/02/2021). The patients/participants provided their written informed consent to participate in this study.

## Author contributions

G-NP, GD, MAt, and JR: conceptualization. GD, JR, EB, and VS: methodology. VS: software. VS, MAt, G-NP, and GD: validation. VS, MAt, and JR: formal analysis. G-NP, MAt, SS, MK, AM, MAn, NZ, and GD: investigation. G-NP, MAt, SS, MK, AK, AM, MAn, NZ, and GD: resources. VS, MAt, GD, and JR: data curation. G-NP, MAt, EB, and SG: writing—original draft preparation. G-NP, MAt, EB, SG, and JR: writing—review and editing. G-NP, MAt, GD, and JR: visualization. GD, JR, and ID: supervision. GD, JR, and MAt: project administration. All authors read and agreed to the published version of the manuscript.
